# Findings from a pilot randomised trial of a social network self-management intervention in COPD

**DOI:** 10.1186/s12890-020-1130-1

**Published:** 2020-06-08

**Authors:** Lindsay Welch, Rosanna Orlando, Sharon X. Lin, Ivaylo Vassilev, Anne Rogers

**Affiliations:** 1 NIHR Wessex CLARHC, Southampton, UK; 2 University of Southampton, Faculty of Environmental and Life Sciences, School of Health Sciences, Building 67, University Road, Southampton, SO17 1BJ UK; 3 Solent University, School of Sport, Health and Social Sciences, RM 126, East Park Terrace, Southampton, SO14 0YN UK

**Keywords:** Chronic Obstructive Pulmonary Disease (COPD), Self-management support, Social intervention

## Abstract

**Background:**

Self-Management Support (SMS), refers to the actions taken by individuals to recognise and manage their own health. It is increasingly recognised that individuals with chronic obstructive pulmonary disease (COPD) require additional support with their Self-management. Emerging evidence suggests that the use of a social network intervention can improve health outcomes and increase quality of life. In order to understand the potential benefits of SMS in COPD, the GENIE (Generating Engagement in Network Support) SMS tool was implemented and evaluated in a COPD primary care context. The GENIE intervention is a social networking tool that consists of 3 parts; a concentric circle modelling to map existing social networks; a questions sections to elicit preferences for activities; a map of selected resources is then produced, aligned with the user’s interests and suggestions for connections to existing network members and to new resources.

**Methods:**

A pilot, parallel, single blind, block randomised controlled trial. Patients with COPD ranging from mild-very severe were recruited. Participants provided written consent and were then randomised to either the intervention or usual care. The primary aim was to understand the clinical benefit through the analysis of health status, symptom burden and quality of life. The secondary outcome measure was health utilisation. NHS cost differences were reported between groups using the GENIE intervention over usual care.

**Results:**

The GENIE pilot results demonstrate maintenance in health status and clinical symptoms with a decrease in anxiety. An overall increase in quality of life was observed, these findings did not reach significance. A cost reduction was demonstrated in inpatient stay with no difference in primary care costs. Overall a cost reduction in NHS service utilisation was indicated in the intervention group.

**Conclusion:**

This pilot study indicated that using a social network intervention can encourage the development of new social connections and extend existing support networks for COPD patients. Increasing network support in this population is of benefit to both patients and NHS providers in terms of cost reductions and enhancing wellbeing. This broadens the understanding of possible new approaches to SMS in community COPD patients, which could now be investigated in a larger population over a longer period.

**Trial registration:**

Clinical Trials.gov PRS National Library of Medicine. Protocol ID number: 19175, Clinical Trial ID: NCT02935452.

## Background

Chronic obstructive pulmonary disease (COPD) is a progressive, life limiting condition, clinically characterised by airflow limitation, sputum hypersecretion and persistent breathlessness [1]. These enduring daily symptoms have a further impact on mobility, nutritional intake and mental health. The disease is further complicated by ‘exacerbations’ or worsening of symptoms and compounded by multi-morbidity [2, 3]. As COPD prevalence and the related social burden grow, this in turn has a multifactorial impact of society, families, and health systems. This is further augmented by deprivation [4], poor housing, continued tobacco use and poor literacy levels, often prevalent in COPD [5, 6] [7].

Traditional approaches to COPD management have focused on clinical pharmacological approaches alongside self-directed action planning to manage exacerbations [8, 9], or therapies such as pulmonary rehabilitation to maintain muscle strength, increase mobility, and manage breathlessness. Pulmonary rehabilitation is a clinically proven and well evidenced intervention [10]. However, post pulmonary rehabilitation access to further community resources are required in order to continue any positive behaviour changes, activity or the peer support that is encompassed by the six-week intervention. Furthermore, a longer-term solution is required to sustain the personal self-management activities, required by individuals to manage everyday life and to maintain wellbeing with concurrent COPD symptoms [11].

In this paper we use the concept of socially supported self-management (SMS) in COPD. An approach designed to shift the focus to a patient centred care model, through the prioritisation of the psycho-social management needs, which implicates links with other people and range of resources to support illness and wellbeing activities. Socially supportive self-management works on the premise of a ‘whole systems model’ of self-management [12, 13].

In order to develop a social approach to SMS support, the nature and purpose of existing social networks should be considered. Social networks are comprised of social ties, these include both strong and weak ties - strong ties consist mainly of family and close relations who are seen frequently and viewed as core informal care givers, who provide the most support. Other connections with less intense involvement with the person (weak ties) such as neighbours or people running groups in the community also have a place in long team illness management in so far as diverse social contacts provide the potential for providing sources of support that can enhance health and wellbeing [14].

The current mainstream delivery of SMS is through educational programmes focussed on action plans to manage symptom flare ups. These plans address the physical aspects of long-term illness management in COPD but often, do not tackle the complexities of day to day living with breathlessness or sustaining wellbeing. In contrast a social network approach works with the recognition that patients with long-term health conditions spend relatively little time in contact with health professionals, in comparison to the time they spend managing their disease alone, or with their family. Therefore, this requires a focus on the connections and the activities needed to manage their condition in day to day life [15]. Building and linking to social network support can potentially draw in a broader set of resources (e.g. exercise, group activities), which can support individuals to manage their life around a complex condition and gain meaningful connections to others in the community [14, 16]. The Generating Engagement in Network Support (GENIE) tool is a social network intervention designed to broaden network support and diversify existing networks [14]. GENIE is based around network mapping, user centred preference elicitation and needs assessment.

The use of GENIE as a social network approach has been evaluated in relation to other long term conditions such as diabetes, chronic kidney disease and mental health conditions [15, 17, 18]. These studies demonstrated positive results in terms of the tools impact on NHS costs and patient outcomes. These included; improved blood pressure control, improvement in quality of life and the uptake of new activities identified through the GENIE tool [19]. The difficulties of managing breathlessness, frequent exacerbations, and a declining disease trajectory pose challenges for people suffering from COPD. The latter warrants conducting a pilot study to ascertain the potential benefits of the application of a socially supported SMS tool in the respiratory population. This study will build an understanding of the value and impact on health care use of a social network intervention for the improvement of SMS for people with COPD.

In this pilot study, we hypothesised that, in line with social environmental approaches such as social prescribing [20] the use of a social network intervention (GENIE), in people with COPD, would address personal social needs through enlisting network support in an engaging and user friendly way. In turn, this could influence long term behaviour change through participation in valued activities, and provide long term SMS through the enlistment of wider resources and links to personal networks. Therefore, the main aim here is to test this approach in a pilot study with people who have COPD a local primary care context.

## Methods

### Study objectives

The study was designed as a pilot study. A pilot study was selected to understand if the main components of the study design work well together [21]. The main aim here is and to answer the research question: to understand the potential clinical and financial benefits of increasing long term health care management options, through a social network approach, in a primary care context, using the GENIE network intervention to build social capacity to support self-management.

The study objectives are: (1) to use social network mapping techniques, activity and resources preferences to engage participants in considering their current support preferences and further needs. (2) To engage participants in wider social activities and linked health resources. (3) To clinically evaluate patient reported symptom improvement in COPD patients using the intervention in comparison to the usual care control group. (4) To evaluate quality of life in participants using the GENIE intervention in comparison to the usual care group and (5) to review health utilisation and relative cost in the GENIE intervention with a view to upscaling for broader application.

The study was delivered in the local community COPD team. The team was a mix of clinicians, physiotherapists, nurses and medical consultants delivering care to COPD patients in a deprived inner-city area falling within the 20–30% decile of deprivation [22], with a known higher than average prevalence of COPD [22]. This area was purposefully selected as deprivation is associated with: isolation, poor health literacy; poor access to health resources, information and sources of influences; insufficient social capital; low personal confidence and higher differentials in power with professionals [23].

The clinical team invited patients to participate in the study either towards the end of their 6-week pulmonary rehabilitation classes or at the review appointment at the end of the programme, after the completion of pulmonary rehabilitation. Researcher (LW) was also a clinician in the team and was involved in offering the study to PR patients.

### Study design

The study follow-up phase was 3 months. Sixty people were recruited from the pulmonary rehabilitation groups. This number was small enough to be delivered in a short time frame, and large enough to have groups for analysis [24].

The aim was to collect health utilisation data reducing the possibility of recall bias (i.e. how many times have you been into hospital over the last 3 months), therefore a longer time frame would require an alternative data collection method to maintain accuracy, such as the diary method.

Block randomisation was used, a commonly used technique in clinical trial design aimed to reduce bias and achieve balance in the allocation of participants to treatment arms, especially in this case when the sample size is small [25]. The sequence was allocated at random in blocks of 4 to ensure an even grouping of participants and to fit with the timing and blocks of consultations. The allocation sequence preselected the random group for the block of 4, so if A = intervention and.

B = control then sequences were selected at random; AABB, ABAB, BABA etc. In order to further reduce unconscious bias of the researcher, the clinical administrative assistant (not included in the research) created pre-prepared envelopes containing the possible combinations of group allocation, that were stored in a locked drawer on site at the health centre.

The research team shared an Excel spread sheet set to generate a random number sequence. The next sequential number will be selected, which will then correspond to an envelope with the predetermined sequence. In this way the envelope selection is random, then sequence in the envelopes in also random, to ensure the 50/50 random grouping of participants.

Socio-economic baseline data was collected, after randomisation included; disease severity (from existing medical records), age, gender, previous employment, educational level, and smoking status and descriptive statistics were then calculated. This data was captured to ensure the study demonstrated a balance of the groups for health literacy and possible existing abilities to understand, socialise and navigate services.

### Ethical considerations

This is a new research trial of an existing social networking tool already used in the management of other long-term conditions, but not yet specifically with COPD. Prior to introducing this to a clinical team, the local NHS research clinical effectiveness group supported the proposal for a pilot study within their organisation.

As this is an intervention within a clinical service and the evaluation was conducted with NHS participants and on NHS properties, therefore University ERGO (Ethics) and HRA full NHS ethics application was sought and granted. Information about the study was provided in an accessible information format, in addition to the usual format for patient information. The study was ethically approved by Hampshire Ethics B REC: 16/SC/0627 and an amendment was passed to enable to use of the revised accessible patient information sheet. Written informed consent was obtained from all participants. The ethical review further evaluated the social and scientific value of the study. Further to this it ensures adequacy of patient information, the informed consent process, recruitment arrangements and access to information.

The service implementation and evaluation were presented at the local NHS clinical effectiveness and audit group and approved, as per local NHS policy. Each participant was offered a patient information sheet and time to take this away and read the information. Written informed consent was gained from each participant either prior to or at the baseline study visit.

### Eligibility

People were eligible to participate if they were over 18 and with a previously confirmed diagnosis of COPD through objective testing, usually by spirometry. There were also required to be currently receiving care through the selected COPD community service. People of all COPD disease severities (categorised by airflow obstruction [26] were included.

**Table:** 

Inclusion Criteria	Exclusion Criteria
• Older than 18 years of age; • Patients with a diagnosis of COPD according with NICE or GOLD guidelines – all severities were included [26] • Currently in the Community COPD team service. • Completed a pulmonary rehabilitation programme in the service in the last 3 months.	• No formal diagnosis of COPD; • Unable to fully express himself/herself in English; • Diagnosis of a mental health condition, or poor cognition. • Extreme anxiety, agitation and/or depression.

### The intervention

Generating Engagement in Network Support (GENIE), is comprised of 4 main stages; each of which work alone or together as a complex intervention. **Picture 1 (see** Additional file 1**).** GENIE has been developed to be an intervention which is co-produced. Co-production in this sense refers to the process of delivery, as the participant themselves builds their own social network and selects their preferences, then retains ownership of the network map and links to favoured activities. The facilitator, who can be from either a lay or professional background, is there to guide the process, not to direct the process. In this way by talking through the GENIE mapping process, individuals can visualise their network. They then have the opportunity to reflect on connections and resources that provide value, and also where there may be gaps in their support. The support can be in the form of social, practical or emotional as well as being specifically related to a health condition, in this case COPD. GENIE’s third stage is the link to the database of locally tailored online and offline groups and resources. The GENIE tool is delivered face to face as part of a consultation, and can be broken down into distinct stages:

- **Stage 1:** Mapping of the individual’s current social support network using a concentric circles method.
- **Stage 2:** Eliciting values and preferences for activities and support resources.
- **Stage 3:** Linking individuals to prioritised and valued activities and resources. (Links are to a pre-created database where local organisations and resources have been categorised).
- **Stage 4:** The GENIE Tool then presents options in a user-friendly way, on a Google map with clear details about access. **Picture 1 (**see Additional file 1**)**

The intervention is usually delivered face-to-face as the facilitated discussion is part of the interventional process. For the purposes of this study the facilitator was the researcher (LW). Researcher LW is a respiratory nurse and the COPD service lead. So was experienced in delivering COPD care. The delivery took 45 min to 1 h.

#### Building community engagement to carry out the evaluation

The GENIE data base needed to contain an appropriate library of social and health activities suitable for local COPD patients. Therefore, the data base was adapted by LW to include links to local exercise groups, social and community activities. The groups included generic actives including; reading, arts and special interest groups. Walking, Tai Chi and more exercise orientated groups or classes. The links and resources were further stratified to include disease specific activities and generic interest groups and clubs. Support for this data base was provided by a local councillor, who had already compiled an offline data base for his constituents. In addition to this the local voluntary Breathe Easy groups, groups designed specifically for peer support with respiratory illness, were enrolled in an Integrated Breathe Easy project, funded by the British Lung Foundation [27]. This provided the groups with professional support to develop as functioning patient groups, and be equipped to accept new people.

#### Briefing the clinical team

In order to successfully implement the GENIE tool into clinical practice; the clinical team were placed at the core of the process in order to ensure appropriate and effective facilitation. Therefore, the local COPD clinical team was briefed at an early stage of the study development and included in the study progress. This was delivered through question and answer sessions and direct learning opportunities in how the tool works and in the objectives of the study.

### Usual care

The aim was to introduce the tool into the local COPD community team, with a view to eventually embedding it as part of an integrated community or primary care clinical consultation. In terms of usual care, the COPD team already had in place a post pulmonary rehabilitation discharge pack. All patients leaving pulmonary rehabilitation received a discharge pack which contained a: British Lung Foundation (BLF) exercise DVD and guidance; advice about local BLF Breathe Easy support groups; and information regarding walking groups. These items were routinely provided either during the rehabilitation programme, or at review at the end of the programme. All of these links were added into the GENIE tool data base to ensure all patients received the same information.

### Outcome measures

The primary outcomes were the participant reported symptom scores, quality of life scores, and social network movement. Secondary outcomes included health care utilisation and health care cost.

### Data collection

Five questionnaires were administered. Clinical symptom questionnaires including the COPD Assessment Tool (CAT), a validated twelve point questionnaire to asses COPD health status [28], the nine point Patient Health Questionnaire (PHQ-9), a validated diagnostic and research tool for depression scoring [29] and the 7-item anxiety scale (GAD-7) for the assessment of generalized anxiety disorder a validated tool for assessing anxiety in clinical practice and research [30]. In addition to the clinical questionnaires, the validated EuroQoL instrument EQ. 5D was administered to describe people’s quality of life in terms of mobility, self-care, usual activities, pain/discomfort and anxiety/depression [31]. Health utilisation data was collected using a healthcare utilisation questionnaire adapted from Client Service Receipt Inventory (CSRI) [32, 33].

Inventory (CSRI) is an economic research instrument developed by Martin Knapp and Jennifer Beecham to collect information on service utilisation, income, accommodation and other cost related variables and is a valid measure [32]. The main purpose of the instrument is to allow resource use patterns to be described and support costs to be estimated using an appropriate unit cost. The instrument was selected for this study to monitor the shift of healthcare usage away from formal care and to investigate the GENIE tools potential abilities to redirect healthcare use away from formal health care activities to voluntary sector resources.

Health utilisation was chosen in preference to admission rates, as it was applicable to a primary care and community context. Furthermore, the utilisation data ‘followed the person’ and the participants were asked to recall their activity over the past 3 months, at baseline and at 3 months follow up. The study measured the participants’ attendance and use of NHS services at baseline and at 3 months cost (primary care, secondary care, community care) and explored the changes before and after using GENIE [34].

These five questionnaires were administered at baseline and re-administered at 3 months follow up post intervention (+/− 2 week). Furthermore, for those in the intervention arm, feedback and discussions were initiated using the network diagrams. Participants were asked again for their permission to record these discussions to ensure parity and quality assurance between researchers.

### Analysis

The study analysed data using SPSS and R, from the intervention arm and the non-intervention arm at baseline and at 3 months post intervention. The findings were compared between the groups at baseline and 3 months post intervention. Clinical comparative analysis of the CAT score, the PHQ-9.

GAD-7 scores and EQ. 5D score pre and post intervention was analysed using Wilcoxon Ranked tests.

Pre intervention symptom scores and quality of life were then correlated with the follow up score (+/− 1 week) symptom scores and quality of life, with significance. These were compared at baseline and at 3 month follow up in both groups, as well as the differences between the two groups [35]. Since these variables under investigation are not normally-distributed, the nonparametric Mann-Whitney test was used to detect the significant difference (before and after) and between the two groups for intervention effect. A 5% cut-off point (*p* = 0.05) was used for significance.

Health service utilisation was captured using a modified version of the CSRI [32]. Unit cost was attached to each data entry, an average value was computed at each label costing. The sample size of 60 participants was too small to extrapolate enough longitudinal data to measure quality adjusted life years. Therefore, the costing trend was reported both across groups and pre and post intervention.

Participant uptake of the social activities was recorded on paper using the Genie tool and compared between groups using network typology counts. Network counts have been previously identified and described by Vassilev in 2018. Networks types were defined by the number of members and frequency of association [36].

## Results

### Study flow and baseline characteristics

Patients were recruited from an inner city region, containing the areas of highest deprivation [37] and COPD prevalence [22].

The study was delivered over 6 months and participants were enrolled for 3 months. Three months was chosen as this relatively short time frame would reduce the risk of recall bias.

**Fig. 1:**
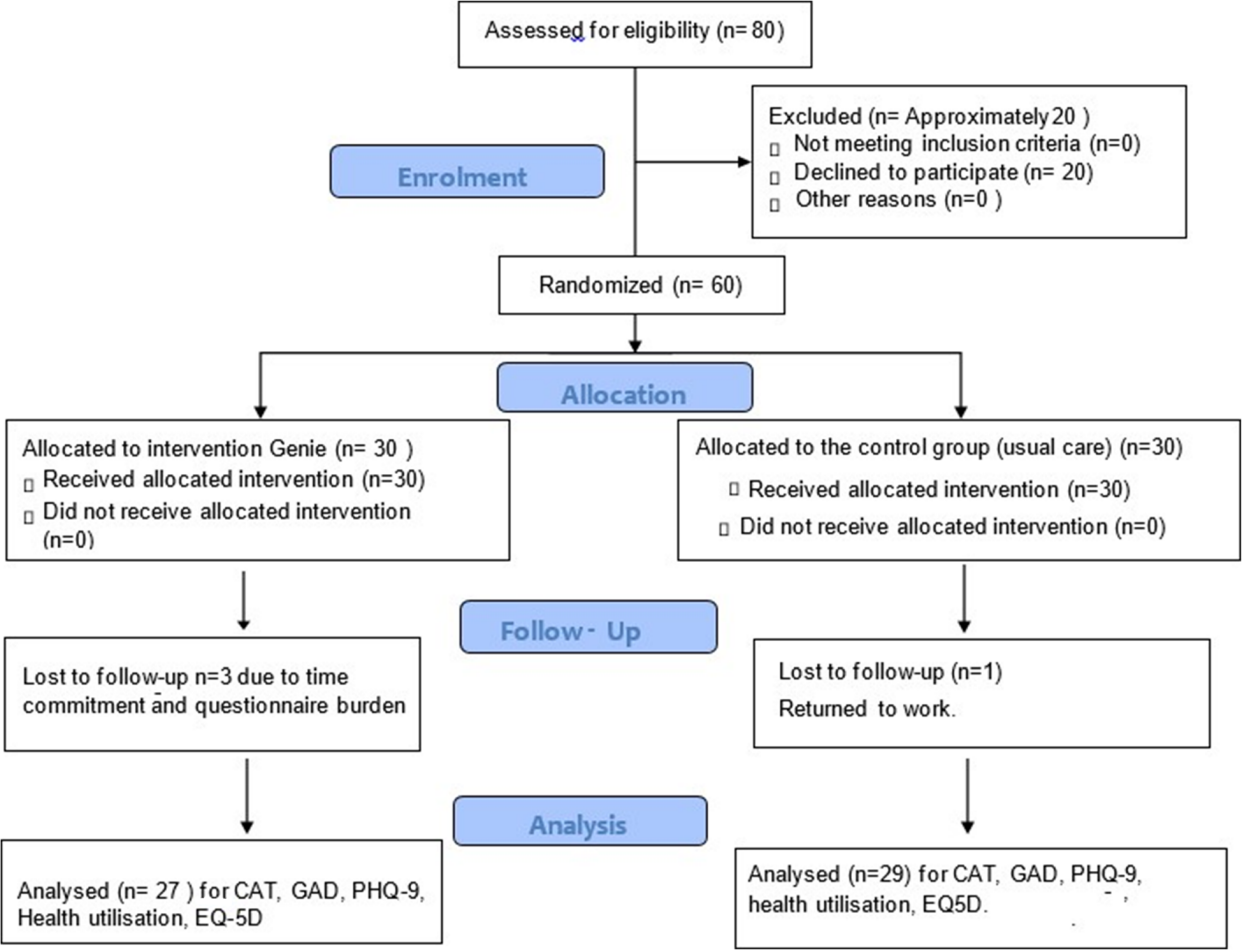
Study consort diagram

Eligibility for the study was assessed throughout pulmonary rehabilitation. However 20 people declined to participate. The reasons included; time commitments (feeling they had already dedicated time to PR), and concerns about participating in a research study. These concerns did prompted a study amendment, to include a more accessible information sheet. This then encouraged more people to enrol, as the information was tailored to be suitable for all readers, and to address any concerns around study participation. During the 3 months study period, three people dropped out of the intervention arm; this was due to time commitments, questionnaire burden and overall frailty. One person dropped out of the control arm because of returning to work. However the study design did allow for an increase in recruitment; however as a pilot study this was maintained at 60 participants (Fig. 1).

The baseline characteristics of the study showed that both groups were broadly similar in respect of age, sex and disease severity, with no statistically significant differences. The mean age in the intervention group was 68.87 and 71.87 in the control arm (not significant). At least half the participants in each arm lived alone. However, a higher percentage of people in the intervention group (83.2%) had one or more regular visitors. In comparison 66.6% of people in the control group received regular visitors (Table 1).

The groups were matched in terms of qualifications and previous employment. There are slightly more smokers in the interventional arm (23% in the interventional arm and 13.3% in the control arm), however mean lung function or COPD severity was matched across the groups, slightly more patients with severe disease in control group, however this is moderated with 26.6% intervention, and 13.3% control in the mild group. The moderate group was more balanced with 36.6% vs 30% respectively, 23.33% vs 40% in the severe group, and 13.33% vs 10% in the very severe groups.

**Table 1:** Baseline Characteristics of the study cohort - Genie in COPD

CHARACTERISTIC	INTERVENTION GROUP (A) ***N*** = 30	CONTROL GROUP (B) ***N*** = 30	***P***- Value***
**GENDER**	50% female	50% female	–
**MEAN AGE**^**b**^	69 ± 6.33	72 ± 8.04	0.11
**HOUSEHOLD COMPOSITION**			0.24
**LIVES ALONE**	16 (53.3%)	14 (46.6%)	
**2 PEOPLE**	14 (46.6%)	12 (40%)	
**3–4 PEOPLE**	1 (3%)	3 (10%)	
**INFORMAL CARE RECEIVED AT HOME**	2(6%)	3(10%)	
**NUMBER OF REGULAR VISITORS**^**a**^			0.21
**0**	5 (16.6%)	10 (33.3%)	
**1–5**	19 (63.3%)	12 (40%)	
**6–10**	4 (13.33%)	6 (20%)	
**10+**	2 (6.6%)	2 (6.6%)	
**QUALIFICATIONS**			0.21
**NO QUALIFICATIONS**	12 (40%)	9 (30%)	
**SCHOOL LEAVING ONLY**	5 (16.6%)	6 (20%)	
**POST SCHOOL EDUCATION (VOCATIONAL)**	9 (30%)	12 (40%)	
**UNIVERSITY / HIGHER EDUCATION**	4 (13.33%)	3 (10%)	
**PREVIOUS EMPLOYMENT**			**–**
**MANUAL**	18 (60%)	18 (60%)	
**SKILLED**	12 (40%)	12 (40%)	
**SMOKING AND COPD**			
**CURRENT SMOKER**	7 (23%)	4 (13.3%)	0.20
**EX SMOKER**	23 (76%)	25 (83%)	
**NEVER SMOKER**	0	1 (3%)	
**MEAN FEV1**^**b**^	0.56 ± 0.23	0.51 ± 0.21	
**COPD SEVERITY*(ONLY 29 IN DATA SET FOR CONTROLGROUP)***			0.21
**MILD**	8 (26.6%)	5 (16.66%)	
**MOD**	11 (36.6%)	9 (30%)	
**SEVERE**	7 (23.33%)	12 (40%)	
**VERY SEVERE**	4 (13.33%)	3 (10%)	

Full data set is available for all baseline data- apart from lung function for 1 participant in the control arm. All other baseline data is complete

*** *P*-values are Pearson’s chi-squared test results except variable “Age” where t-test is used

^a^no of family or friends visiting daily to weekly

^b^Mean ± Standard Deviation

### Social network outcomes

Network analysis identified the type of social network at baseline, and then compared this at the follow up visit. Unfortunately, a small number of people declined to repeat the concentric circle mapping exercise, so this reduced the number of networks analysed to 27, rather than the original 30 participants. Network types and characteristics have been linked to the capacity of inter-personal environments to mobilise and share resources [36]. Therefore, the wider and more diverse the network the increased potential to negotiate increased support and resources from these avenues.

In this case we are reporting the extension of networks; network type, any additional new activities [1], both online and offline [2]. Engagement in networks is also reported in terms of personal reflection of existing network support [3], increased frequency of contact [4], and additional network members [5]; scored out of 5 (Table 2).

**Table 2:** Network changes. Network Changes at Time 2 = 3 months post intervention (*n* = 27–3 dropped out)

Baseline		Extending Networks			Network engagement				
Study Participa nt Number	Type of network At Baseline	Additional new activities	Type of network at 3 months follow up	Online	Reflection on existing support	Increased contact with existing groups/people	Additional new network members		Change within network Score
002	Family centred	0	family Centred	0	1	1	1	Addition of less frequent contact with distance relatives and a new close friend, seen at least once a week.	3/5
003	Family Centred	*0*	*Declined to repeat the social circles*	0	0	0	0	n/a	0/5
005	Very Small network (4 members only)	0	Very Small network (4 members only)	0	0	0	0	Very severe COPD and hospitalised during the study.	0
008	Small (8 members)	0	Small (8 members)	0	0	0	0	Very severe COPD and hospitalised during the study.	0
009	Small (8 members)	0	Friend supported	0	1	1	1	4 additional network members, greater involvement with the church, reduction in frequency of seeing health providers.	3/5
014	Family centred (18 members)	0	Family centred (18 members)	0	0	0	0	Carer or her grandchildren, no capacity to diversify.	0
015	Very small	0	Very small	0	0	0	0	Did not wish to diversify.	0
018	Diverse	0	Diverse	0	0	0	0	Already diverse.	0
019	Diverse	0	Diverse	0	1	1	1	Movement post intervention – 2 people left the group, 2 people joined, 3 people became closer and more valued. More value and time attributed to groups.	3/5
021	Very Diverse	0	Very diverse	0	0	0	0	No movement already very diverse network	0
022	Family Centred	0	Friend Supported	0	1	1	0	Valued friends became closer as their support was helpful.	2/5
025	Very small	1	Diverse	0	1	0	0	Joined 3 weekly health related groups	2/5
027	Diverse	0	Diverse	0	1	0	0	Maintained groups and friends- but saw less of her daughter and son in law	1/5
030	Family Centred	1	Diverse	0	1	1	1	Addition of 3 more friends/ neighbours and joined 2 groups.	4/5
033	Friend centred	1	Diverse	0	1	1	0	Increased number of groups and group frequency	3/5
037	Family supported	*0*	*Declined follow up*	0	0	0	0	n/a	0
040	Family Centred	0	Family centred	0	0	0	0	No change- had been unwell in the past 3 months since GENIE	0
041	Family and Friend Centred	0	Family and Friend Centred	0	0	0	0	Declined to discuss at follow up.	0
043	Small	1	Family and Friend Supported	0	1	1	0	Increase in regular friends visiting, and joined an exercise group.	3/5
045	Family Centred	0	*Declined follow up*	0	0	0	0	n/a	0
047	Family Centred	0	Family Centred	0	0	0	0	No change as admission and been unwell over the 3 months follow up period.	0
050	Small	1	small	1	1	0	0	However, this person liked to have a small network due to social anxiety and supported the setup of groups for others and joined a new exercise group	3/5
052	Friend Centred	0	Friend Centred	0	1	1	0	No change – movement in friends – but within the existing network.	2/5
053	Friend Centred	0	Friend Centred	0	1	1	0	Increased health engagement with services and decreased family reliance, however remained in a friend centric network.	2/5
055	Family Centred	0	Family Centred	0	0	0	0	Awaiting lung transplant, currently reliant on family.	0
058	Friend and family supported	1	Diverse	0	1	1	0	Many peripheral friends and addition of 2 health related groups	3/5
060	Friend Centred	0	Diverse	0	1	1	1	Increase of frequency of group meeting and addition of 2 friends	3/5

Overall 11 people increased their network frequency and members enough to move up a group. 13 people remained the same. Note that 4 of these people already had diverse or very diverse networks. Time 2 refers to changes 3 months after the intervention

In terms of network movement 15 people (55%) increased network members, frequency of social interactions, online engagement, and reflection or engaged in additional activities. 12 people (44%) did not further diversify their networks. Therefore, a higher proportion of people increased engagement or activity extended their network.

Networks have been scored out of 5, those over 3 have reflected on the input they are receiving from existing social networks, and have made changes, within their capacity to do so. Many people were unwell during the study, and therefore did not have the capacity to take on additional activity. The potential impact of acute fluctuations of long-term illness are important considerations for future development of this work.

The social movements are a positive reflection of the changes realised after using the intervention and the value of the concentric circle discussion.

Overall 55% of the participants increased their social networks and 44% of the participants remained in the same network category. The stability and increase in networks are both positive outcomes in a population who struggles to maintain links and negotiate resources in times of adversity (ill health and declining disease trajectory) [36].

### Clinical outcomes

Changes in clinical symptom data and quality of life were expressed by the median and the interquartile range (IQR) of each variable (Table 3).

**Table 3:** Full table of outcome variables Genie in COPD **(median, IQR in square brackets)**

Outcome	Intervention group Pre	Intervention Group Post	Intervention group Difference (Post-pre)	Control group Pre	Control group Post	Control group Difference (post-pre)	Diff in Diff ^a^	Significance
	Baseline	Follow up		Baseline	Follow up			
CAT	21 [16, 26]	21 [16, 24]	-1 [−5, 2]	18 [10, 22]	15 [10, 25]	0 [−3, 1]	349	*p* = 0.49
GAD-7	2.5 [1.0, 8.8]	3.5 [1.0, 5.0]	−0.5 [− 3.5, 0.8]	2.5 [1.0, 5.8]	4.0 [1.0, 6.0]	0.0 [−1.0, 3.0]	280	*p* = 0.10
PHQ9	5.5 [3.0, 12.8]	5.5 [2.5, 10.0]	−1 [−3.0, 2.0]	4.0 [2.0, 6.8]	4.0 [1.0, 8.0]	0.0 [−1.0, 2.0]	317	*p* = 0.32
EQ-5D	0.56 [0.37, 0.71]	0.62 [0.53, 0.69]	0.04 [−0.08, 0.21]	0.64 [0.45, 0.76]	0.67 [0.39, 0.77]	−0.001 [− 0.18, 0.10]	484	*P* = 0.13

^a^Mann Whitney test is used to calculate control group and intervention group differences in the differences present before and after the intervention

The measure of overall symptom burden in COPD - the CAT score - remained stable in the intervention group but increased in the control group (demonstrating a higher symptom burden). The Generalised Anxiety Score (GAD) score decreased in the intervention group by a marginally significant difference (*p* = 0.1) to that in the usual care group where a rise in anxiety symptoms was recorded. The PHQ-9 score declined slightly in the intervention group (representative of improved symptoms), although not significant from that of the control group, − 1 decrease in score in the intervention group with no increase in the control group.

The overall quality of life measured by the EQ-5D showed improvements of 0.04 in the intervention arm over the 3 months and a fall in quality of life in the usual care arm of − 0.001 (*p* = 0.13).

Overall these findings are not statistically significant but the trend is in favour of the intervention group in a reduction in anxiety scores.

### Health utilisation outcomes

Health utilisation questionnaire was grouped into subsections (inpatient, outpatient, primary care, community-based specialist or generic health care, or accessing their social worker, if applicable). It was administered twice, at baseline and at 3 months, in order to track changes over time. The Unit Costs of Health and Social Care report [38] was used, as this is a nationally approved and applicable source of data. This compendium is produced annually by the Personal Social Services Research Unit [38]. Inpatient data was costed using National Reference Cost [39].

**Table 4:** Costs/savings to the NHS

	Intervention			Control			
	Pre-Genie	Post- Genie	Difference intervention	Pre-Genie	Post- Genie	Difference control	Difference of difference Favour of I or C
Surgery GP	£ 1,731	£1,910	+£180	£1,509	£2,763	+£1,254	£1,075 (I)
Surgery Nurse	£ 333	£ 263	-£70	£ 292	£ 436	-£144	£14 (C)
Community Care	£ 935	£ 914	-£21	£ 734	£ 683	-£51	£30 (I)
Outpatient	£ 2,544	£ 1,149	-£1,395	£ 1,845	£ 3,575	+£1,730	£3,125 (I)
Inpatient	£ 13,834	£ 7,600	-£6,234	£ 6,871	£ 3,892	-£2,979	£3,255 (I)
Others (PR, maintenance)	£ 145	£ 240	+£95	£ 80	£ 68	-£ 12	£107 (C)
Total NHS cost/savings	£ 19,653	£ 12,019	-£7,634	£ 11,490	£11,417	-£73	£7,561 (I)
Band 8 cost (3 months/30 patients)	Total cost over 3 months= **£2,820** Intervention cost per patient when delivered by a Band 8 = **£94.00**						
Total cost/savings to the NHS	Savings to the NHS in 3 months in the intervention group Genie delivered by a band 8a = **£4,814**						
Saving per patient to the NHS	Savings of £160.00 per patient						
Band 3 cost (3 months/30 patients)	Total cost over 3 months = **£1,050** Intervention cost per patient when delivered by a Band 3 = **£35**						
Total cost/savings to the NHS	Potential savings in 30 people over 3 months if the intervention is delivered by a band 3 = **£6,584**						
Saving per patient to the NHS	Savings of £220.00 per patient						

The cost of intervention per patient is £94, if Genie is administered by a senior community nurse: Agenda for Change (AFC) Band 8 while for future implementation the cost would be £35 per patient considering an AFC band 3 NHS colleague. Genie is user-friendly and versatile and can be used without health professional guidance, after familiarisation with the tool Table 4.

Health utilisation in the general COPD population is expected to increase over time, due to the continuing illness trajectory and health deterioration [40]. Therefore, an overall rise in health care costs would be expected in both arms (intervention and usual care). The trend of costs before and after Genie counts of 40% reduction in costs, in the intervention group, with a margin of £7634. While in the control group the reduction is less than 1%. Costing breakdown comparison in (Table 4).

The drivers of this dropping cost are inpatient activity and outpatient visits, this accounts around £6234 and £1395 respectively in the intervention group over 3 months.

The control arm counts also indicate dropping inpatient activity, which is reported to be around £2979. Although outpatient and GP visits increased within the 3 months with a marginal value of around £1730 and £1254 respectively. This cost difference is minimal largely because outpatient visits are pre-planned (Table 4).

Considering that COPD, is a progressive condition, requiring increasing primary care support throughout the illness trajectory then the aim would be to promote stability in cost of GP interactions, or a slower increase in than the control arm. Therefore, the increase of £180 for GP visits within 3 months in the intervention group can be considered as a positive effect in favour of the intervention. In order to calculate cost effectiveness a larger sample size would be required with a longer follow-up time.

## Discussion

A social network tool (GENIE) has the potential in terms of engaging people and acting as a complimentary addition to existing clinical management options in COPD. It was hypothesised that using a social intervention would reframe SMS in COPD and address personal social needs through enlisting peer support in a positive pro-active way. In this respect the intervention appears to have had positive results.

The intervention study further aimed to build a continuum of support with self-directed care after discharge from statutory NHS services. This continuum envisaged through the development of a peer supported community care pathway, which links directly to NHS services. In this way utilising the skills and knowledge of peer networks to monitor and provide simple advice if and when health status deteriorates. Furthermore, people within the peer networks can motivate each other to continue to access voluntary and community resources in order to maintain wellbeing and prevent chronic disease decline over time. This is displayed through the increases in social network reflection and the ability to enlist support and develop new social connections.

Social networking has been utilised and examined in other long term conditions, in particular chronic kidney disease (CKD) [19]. The CKD study used telephone guided access to community resources in primary care. The intervention was referred to as patient-led assessment for network support (PLANS) or the BRIGHT intervention. The intervention utilised tailored information (via the telephone) to sign-post patients to community resources. The intervention was also modestly successful in terms of improvement of blood pressure control and improvements to health related quality of life. However these did not directly translate into increased active engagement in life. In comparison this study (GENIE in COPD) was delivered as a face to face facilitated intervention, rather than telephone guided. Within this study the GENIE tool achieved stability on clinical outcomes, rather than measurable improvement in clinical outcomes. However, both studies support the approach of social networking as a tool to support self-management.

The clinical stability was demonstrated by the consistency in clinical outcome measures, and a small reduction in anxiety in the intervention group. It is noted however that the reduction in anxiety scores and quality of life were not statistically significant, but trended towards the GENIE intervention in a small population of people in a short time frame.

Moreover, by embedding the delivery of the intervention within the clinical team this added further significance in terms of access to community resources for clinicians. Prior to this study voluntary groups for respiratory disease were limited in number and accessibility. The joint working with the British Lung Foundation, local councillors within the community COPD team enhanced care through the promotion of the concepts of network use and valued activities. Therefore, by enhancing and maintaining social networks have in this case demonstrated a pressure reduction in NHS resources in terms of utilisation and cost through socially supportive self-management activity. A second paper [41] describes the process evaluation; including fidelity, reach and dose and discusses the value of the GENIE intervention as an adjunct to supporting long term health behaviour change.

The study processes were acceptable for use in a larger randomised clinical trial, as participants were able to use the tools and complete the questionnaires, the clinical team were engaged and felt confident to support recruitment to the study. A larger scale trial has already begun to address social isolation in the local communities across the whole city. This study is broadly inclusive of any population that could be isolated due to long term illness or social circumstances, not specifically related to people with COPD.

Furthermore, as a positive consequence of the study the COPD clinical service has shifted in its focus to embrace the more patient centred approach of understanding the valued activities and social worlds of COPD patients within their care.

### Limitations

Overall the study was successful, and participants were pleased to be included in an intervention post pulmonary rehabilitation completion. The study was designed to collect clinical data at the study baseline. However, a limitation of this data collection was not accounting for the prior clinically significant improvements during the participant’s recent PR programme. The correlation of these clinical figures may have provided an indication of those people who may have continued to improve in the GENIE programme. Including the PR data in future study design would be beneficial.

However, the nature of recruiting from groups, did lead to peer discussion about the study. Much of this was positive, and encouraged others to volunteer, however some of the peer discussion did lead to some contamination in the randomisation process. However, this was not significant. A larger sample size would have benefitted the economic evaluation and enabled a more robust statistical evaluation, such as a cost effectiveness, or cost benefit analysis. The participants included in the study often struggled with their social and economic circumstances had poor literacy levels and therefore had difficulties with comprehension of the study literature. Therefore, an ethical amendment was required to change the patient facing literature during the recruitment phase of the study. Furthermore, the short follow up period was designed to maintain the ability to recall health use. However, some participants found it difficult to remember activity regarding self-reported health service utilisation, so there is variability in the accuracy of this data due to this potential recall bias. This could have been supported by using a diary method of data collection, or an online data capture device, to improve participant recall.

The short time frame and lack of qualitative ethical approval further limited the depth and length of data capture to only 3 months. Mixed methods could have enhanced the social network discussion and the generation of qualitative data could have supported the figures in terms of network analysis. In this was providing greater depth and understanding in terms of socialisation and support needs of the participants. The study could have been further enhanced by including an outcome measure to review confidence and self-care skills of the study participants; such as the Patient Activation Measure [42].

### Implications for clinical practice and commissioning

The pilot study suggests that the GENIE social networking intervention could have a positive impact on quality of life, anxiety, and on health care utilisation, and the way people approach and use NHS services.

Therefore, this work could enhance existing community and primary care services to implement and encourage social network engagement, to increase patient confidence and foster peer support to assist complex health related decision making in the community, encouraging safer and appropriate approaches to health care use. Furthermore, GENIE does reduce the costs of health care contacts, by redirecting activity to community and planned GP visits with a reduction in inpatient stay.

The implications for the reduction of NHS costs within the intervention group are of interest, due to the ever-increasing prevalence of COPD and reduction in NHS resources. The future challenge is to ensure the GENIE tool is located at appropriate points in NHS services to ensure appropriate and safe care is provided, and the GENIE intervention serves as a tool to build social capital, community resources and the ability to navigate local support networks in clinical settings. Notably **self-management** is something that most patients with a long-term condition have to do every day- not only when they are unwell. The GENIE tool has been used in other long term conditions, diabetes and kidney disease, and has been implemented on the Isle of Wight, and in areas in Manchester. The concept of social network support is broadly adaptable to any language or region. However local, appropriate and accessible activities will need to be added to the database, to ensure regional and cultural specificity.

The GENIE tool can effectively signpost to resource’s online and offline to initiate peer and social support to work through daily solutions to manage long term conditions. These peer ‘top tips’ – are often more valuable than clinical insight. As many people struggling with implementing regimes into their day to day lives.

## Conclusion

The GENIE tool, a social network intervention has yielded positive outcomes in reduction in health utilisation costs and network engagement. This study begins to broaden the understanding of possible new approaches to how to encourage and use social support networks in community COPD patients. Which could now be investigated in a larger population of people with COPD for a longer period.

## Supplementary information

**Additional file 1.** Picture 1.

## Data Availability

The study data and materials have been made available via the Dryad data repository: Welch, Lindsay et al. (2020), Data from: Findings from a pilot randomised trial of a social network self-management intervention in COPD, v3, Dryad, Dataset, 10.5061/dryad.2mn5v02 A further clean copy of the data sheet has been appended to this submission. The raw data is also available upon reasonable request.

## Abbreviations

SMS: Self-Management Support
COPD: Chronic Obstructive Pulmonary Disease
GENIE: Generating Engagement in Network Support
BLF: British Lung Foundation
NICE: National Institute of Clinical Excellence
GOLD: Global Initiative for Chronic Obstructive Lung Disease
CAT: COPD Assessment Tool
PHQ-9: Patient Health Questionnaire
GAD-7: Generalised Anxiety Disorder
EQ 5 D: EuroQoL instrument
CSRI: Client Service Receipt Inventory.
ERGO: Ethics Research Governance Online.
HRA: Health Research Authority.
PSSRU: Personal Social Services Research Unit.
AFC: Agenda for Change.
