# Supplementary material for: Findings from a pilot randomised trial of a social network self-management intervention in COPD
Source: BMC Pulm Med. 2020 Jun 8;20:162. doi: 10.1186/s12890-020-1130-1 (PMC7278059; doi:10.1186/s12890-020-1130-1)

Appendix 1
Picture 1

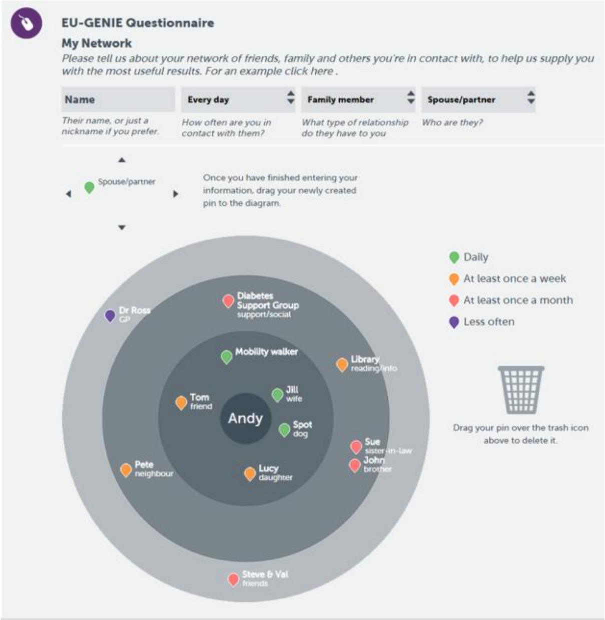

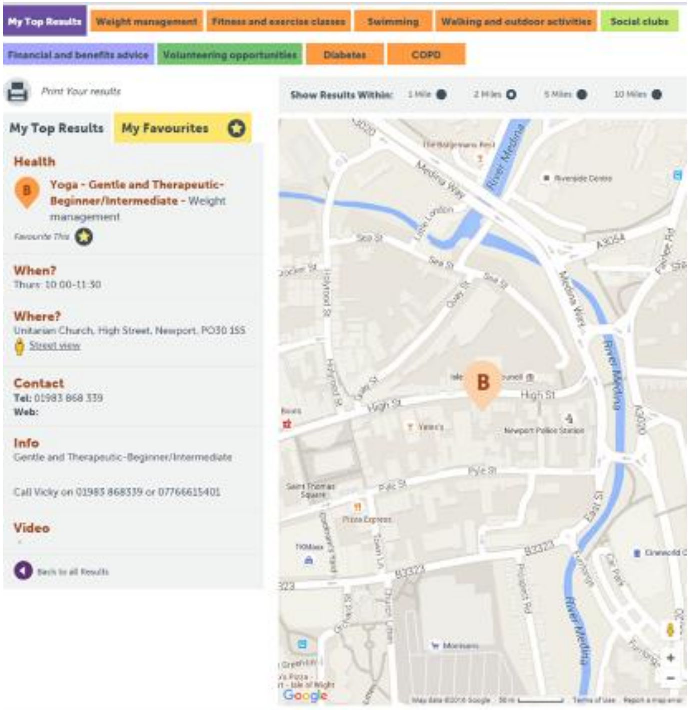

Supplement: Supplementary file 1 — Additional file 1. Picture 1. [file 12890_2020_1130_MOESM1_ESM.pdf]
